# The First Analysis of Synaptonemal Complexes in Jawless Vertebrates: Chromosome Synapsis and Transcription Reactivation at Meiotic Prophase I in the Lamprey *Lampetra fluviatilis* (Petromyzontiformes, Cyclostomata)

**DOI:** 10.3390/life13020501

**Published:** 2023-02-11

**Authors:** Sergey Matveevsky, Nikolay Tropin, Aleksandr Kucheryavyy, Oxana Kolomiets

**Affiliations:** 1 Vavilov Institute of General Genetics, Russian Academy of Sciences, 119991 Moscow, Russia; 2 Vologda Branch of the Russian Federal Research Institute of Fisheries and Oceanography, 160012 Vologda, Russia; 3 Institute of Ecology and Evolution, Russian Academy of Sciences, 119071 Moscow, Russia

**Keywords:** lamprey, cyclostome, meiosis, chromosome, chromatin, histone, RNA polymerase II

## Abstract

Transcription is known to be substage-specific in meiotic prophase I. If transcription is reactivated in the mid pachytene stage in mammals when synapsis is completed, then this process is observed in the zygotene stage in insects. The process of transcriptional reactivation has been studied in a small number of different taxa of invertebrates and vertebrates. Here, for the first time, we investigate synapsis and transcription in prophase I in the European river lamprey *Lampetra fluviatilis* (Petromyzontiformes, Cyclostomata), which is representative of jawless vertebrates that diverged from the main branch of vertebrates between 535 and 462 million years ago. We found that not all chromosomes complete synapsis in telomeric regions. Rounded structures were detected in chromatin and in some synaptonemal complexes, but their nature could not be determined conclusively. An analysis of RNA polymerase II distribution led to the conclusion that transcriptional reactivation in lamprey prophase I is not associated with the completion of chromosome synapsis. Monomethylated histone H3K4 is localized in meiotic chromatin throughout prophase I, and this pattern has not been previously detected in animals. Thus, the findings made it possible to identify synaptic and epigenetic patterns specific to this group and to expand knowledge about chromatin epigenetics in prophase I.

## 1. Introduction

Meiosis refers to two-stage cell division (reduction division: meiosis I; and equational division: meiosis II) accompanied by a range of chromosomal events, including synapsis, recombination, and two rounds of chromosome segregation, the corollary of which is the formation of haploid gametes. Evolutionarily, the meiotic pattern is conserved among eukaryotes [1,2]. More interesting are the differences found among taxa. One of the features of meiosis is the presence of an extensive system of structural and functional chromatin changes. The main discoveries in this field have been found in mammals, for example, and are reflected in several of the papers referred to below.

First, it has been established that a massive rearrangement of three-dimensional chromatin organization occurs in meiosis, implying that chromatin compartmentalization is attenuated to facilitate the expression of spermatogenic genes [3]. Second, prophase I chromatin organization is associated with specific chromosomal interactions that create a special nuclear architecture. These features of meiotic nuclei include the formation (between homologous chromosomes) of special skeletal multiprotein structures (synaptonemal complexes; SCs), to the lengths and types of chromosomes, certain centromere features, specific numbers of heterochromatin regions, and the ability to form chromocenters as well as to the connection of telomeric sites to the nuclear envelope through shelterin and LINC complexes [4,5,6].

Third, the transcriptional chromatin activity differs between stages of meiosis. It is noteworthy that, concurrently with the above-mentioned chromosomal events, a complex gene expression program unfolds. This program involves the expression of more than twenty-thousand transcripts exclusively at different meiotic substages; some of these transcripts are necessary for direct meiotic functioning, and others are necessary for future postmeiotic differentiation [7,8,9,10]. It has been immunocytochemically shown that the transcription level is low in leptotene and zygotene before transcription is reactivated in pachytene, which corresponds to a previously identified sharp switch in global gene expression at this stage [7]. Then, transcription is retained in diplotene and is again suppressed in metaphase I until the end of the second meiotic division [11,12,13]. The transcriptional activity in prophase I is directly related to changes in chromatin structure, particularly to post-translational modifications of specific nuclear proteins (histones), including acetylation, methylation, phosphorylation, ubiquitination, and sumoylation [14]. For instance, asynaptic regions in early prophase I trigger transcriptional chromatin inactivation (meiotic silencing of unsynapsed chromosomes: MSUC) [15], which involves the phosphorylation of H2AX histone on serine 139 in response to the formation of DNA double-strand breaks [16,17,18]. Trimethylated histone H3K9 (H3K9me3) and monomethylated histone H3K4 (H3K4me1) fill the entire nucleus in murine early prophase I and, starting from pachytene, are exclusively retained only in specific small areas [12,13,19], which may indicate their role in meiotic silencing.

Thus, chromatin modification in prophase I is linked with comprehensive programs of gene expression and epigenetic reorganization. The main discoveries in this field have been made in a conventional mouse model. Information on the synchronization of chromosomal synapsis and transcription, as well as the patterns of transcription and meiotic silencing in other taxa of invertebrates and vertebrates, is scarce and fragmentary. Analyses of new species make it possible to test the interdependence of synapsis and transcription reactivation. In this regard, the study of lower vertebrates seems relevant.

In this paper, we present the results of our study on a living “fossil”: the European river lamprey *Lampetra fluviatilis* Linnaeus, 1758 (Petromyzontiformes, Cyclostomata). Migratory lampreys are eel-shaped facultative hematophagous ectoparasites or predators [20,21]. Agnathans separated from the main branch of the vertebrate evolutionary tree, i.e., jawed vertebrates (gnathostomes), between 535 and 462 million years ago [22,23,24]. They formed an evolutionarily independent lineage of lower vertebrates with pronounced features of the lifestyle, morphogenesis, embryogenesis, gametogenesis, and genome. Studies on this animal group are of considerable interest in terms of understanding the early stages of vertebrate divergence [25]. In recent years, hagfish and lampreys attracted increasing attention from researchers owing to a breakthrough in the elucidation of the role and mechanisms of elimination of a substantial part of the genome during normal embryonic development [26,27,28]. Surprisingly, to our knowledge, there is still not a single report on SCs and meiotic processes of the first meiotic prophase in representatives of jawless vertebrates. Here, for the first time, we present detailed immunocytochemical and electron microscopy analyses of chromosome synapsis and transcription in river lamprey prophase I. These experiments allowed us to identify epigenetic patterns specific to this species and to add new pieces of the puzzle to the discussion of the scientific issues under study.

## 2. Materials and Methods

### 2.1. Lampreys

Four adult males of *L. fluviatilis* (LF-01, LF-02, LF-03, and LF-04) were caught in the Chernaya River near the Karshevo village (Pudozhsky District, Karelia, Russia). The Chernaya River belongs to the Onega Lake basin. The studied lamprey is a freshwater potamodromous form of *L. fluviatilis* from the eastern part of the species’ area.

### 2.2. Meiotic Chromosome Studies: Immunostaining Procedure, Controls and Image Analysis

SC preparations were made and fixed according to Peters et al., 1997 [29] with modifications [30] for immunostaining and Kolomiets et al., 2010 [31] for immunostaining and silver nitrate staining.

The slides were washed in phosphate-buffered saline (PBS). Spreads were blocked with HB (holding buffer: PBS, 0.3% bovine serum albumin (BSA), 0.005% Triton X-100). The slides were incubated overnight at 4 °C with primary antibodies: rabbit polyclonal anti-SCP3 (synaptonemal complex protein 3 or SYCP3; #15093, Abcam, Cambridge, UK), mouse monoclonal anti-SCP3 (#97672, #205846, Abcam) diluted to a concentration of 1:200–1:500 in ADB (Antibody Dilution Buffer: PBS, 3% BSA, 0.05% Triton X-100), mouse anti-RNA polymerase II (RNAPII; 1:200, #5408, Abcam), rabbit anti-H3K4me1 (histone H3 monomethylated at lysine 4; 1:300, #8895, Abcam), mouse anti-phospho-histone H2AX (H2A histone family member X or γH2AFX; 1:500, #22551, Abcam), mouse anti-fibrillarin (1:200, #4566, Abcam), mouse anti-H3K9me3 (histone H3 trimethylated at lysine 9; 1:100, #6001, Abcam), human anti-centromere antibodies CREST (calcinosis, Raynaud phenomenon, esophageal dysmotility, sclerodactyly, and telangiectasia; 1:100, #90C-CS1058, Fitzgerald Industries International, Concord, MA, USA) and ACA (anticentromere antibody; 1:100, #15-235, Antibodies Incorporated, Davis, CA, USA), both diluted in ADB. The slides were washed in PBS and incubated with goat anti-rabbit Alexa Fluore 488 conjugated antibodies (1:500, Abcam), goat anti-human Alexa Fluore 546 conjugated antibodies (1:200–1:400) and goat anti-mouse Alexa Fluore 555 conjugated antibodies (1:400) at 37 °C for two hours. The slides were washed with PBS, rinsed briefly with distilled water, dried and mounted in Vectashield with DAPI (4,6-diamidino-2-phenylindole; Vector Laboratories, Burlingame, CA, USA). For some details of immunocytochemistry procedures, see [32,33]. The slides were analyzed with an Axioimager D1 microscope CHROMA filter set (Carl Zeiss, Jena, Germany) equipped with Axiocam HRm CCD camera (Carl Zeiss) and image-processing AxioVision Release 4.6.3 software (Carl Zeiss). Images were processed by Adobe Photoshop CS5 Extended.

The negative control consisted of (1) overnight incubation with a primary antibody (SYCP3, #15093) without any secondary antibodies and (2) overnight incubation with a secondary goat anti-rabbit Alexa Fluore 488 conjugated antibody without primary antibody in a humid chamber. After incubation, the slides were washed in PBS 3 times for 2 min and then embedded in Vectashield medium with DAPI. Then the slides were examined under a microscope using standard exposure. The microscope used fluorescent filters from Carl Zeiss: Filter set 01 (blue), Filter set 38HE (green), and Filter set 43HE (red).

The statistical analysis of all data was performed using GraphPad Prism 9 software (San Diego, CA, USA). Mean values (M) and standard deviation (SD) or standard error of the mean (SEM) were calculated by the descriptive option of the software. The *p*-values reported in Tables S1 and S2 were calculated by Mann–Whitney two-sided non-parametric test. All diagrams were created by graph options of the software.

ImageJ 1.45o software (National Institutes of Health, Bethesda, MD, USA) was used to analyze the images of lamprey spermatocytes, and the average fluorescence intensity of each nucleus (average fluorescence intensity = fluorescence intensity per nucleus area, %, mean ± SEM) was measured accordingly (Table S2). For analysis, we used only the most obvious and clearly visualized (SYCP3 signals) nuclei at the corresponding stages of prophase I. The program took into account the number of zones with a signal (white/gray RNAPII dots) and without a signal (black areas). Analysis of the fluorescent signal intensity was performed starting from the mid zygotene when a sufficient number of RNAPII foci were already distributed through the nucleus.

### 2.3. Electron Microscopy

The slides were stained with 50% silver nitrate solution in a humid chamber at 56 °C for 3 h. The slides were washed in three or four changes of distilled water and air-dried. The stained slides were observed in a light microscope, suitably spread cells were selected, and plastic (Falcon film) circles were cut out with a diamond tap and transferred onto grids. The slides were examined under JEOL JEM 1011 electron microscope (Jeol Ltd., Tokyo, Japan).

## 3. Results

### 3.1. Lamprey Chromosome Synapsis

To investigate the events and processes occurring in prophase I, we used immunostaining of marker proteins. Each stage was described in accordance with generally recognized morphological changes in chromosomes [1,12,34,35].

All spreads were prepared from four unpaired whitish testicles from four males of river lampreys and examined under light fluorescent and electron microscopes. Numerous spermatocytes of varying degrees of spreading were observed on all slides. Due to the large number of chromosomes, many meiocytes were not spread out sufficiently, and there was a lot of chromosome overlap.

**Figure 1:**
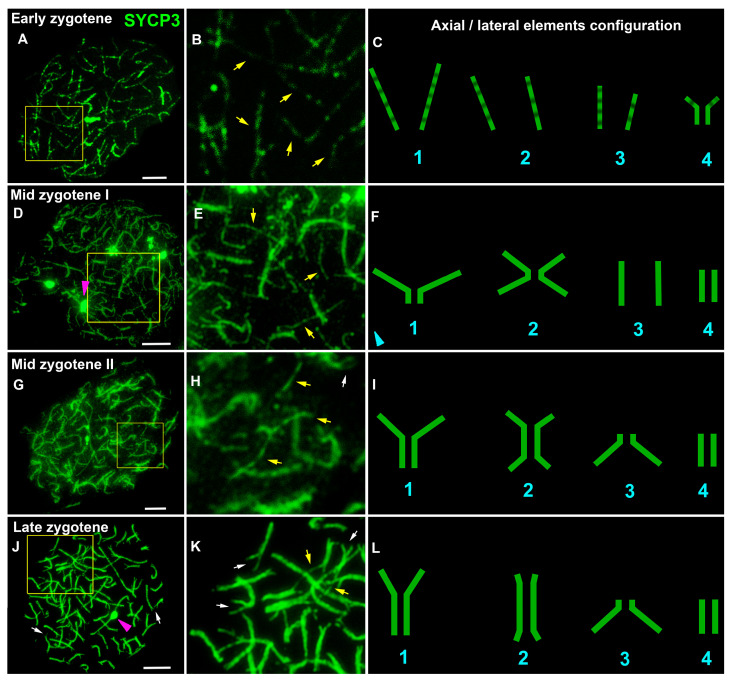
Chromosome synapsis at zygotene substages in the lamprey. SYCP3 staining (green) revealed the structure and behavior of axial and lateral elements of an SC. White arrows indicate asynaptic forks in telomeric regions of SC bivalents. Yellow arrows indicate asynaptic axes. Pink arrowheads indicate SC-associated rounded structures (SCASs). (**A**–**C**) Early zygotene: the presence of thin asynaptic axes and short segments of SCs. (**D**–**I**) Mid zygotene: a large number of thin asynaptic axes, short and long segments of the SC (medium and long chromosomes), or entire SCs (short chromosomes). Other images of this nucleus (**D**) are shown in Figures S1C,C1 and S7A–C. (**J**–**L**) Late zygotene: the presence of areas of interstitial and distal asynapsis and most SC bivalents with asynaptic forks of different lengths and widths. Parts of the images of the nuclei (yellow outline in (**A**,**D**,**G**,**J**)) are enlarged (**B**,**E**,**H**,**K**). Schemes of configurations of axial elements and lateral elements of the SC are presented in (**C**,**F**,**I**,**L**). Scale bar = 5 µm.

**Figure 2:**
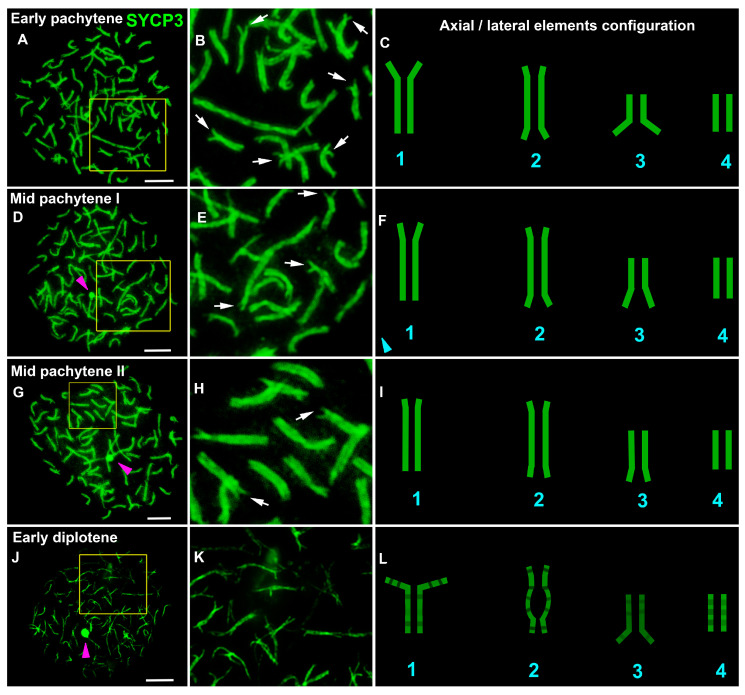
Chromosome synapsis at pachytene and diplotene in the lamprey. SYCP3 staining (green) revealed the structure and behavior of axial and lateral elements of an SC. White arrows indicate asynaptic forks in telomeric regions of SC bivalents. Pink arrowheads indicate SC-associated rounded structures (SCASs). (**A**–**C**) Early pachytene: lack of interstitial and distal asynapsis and most SC bivalents with asynaptic forks of different lengths and widths. (**D**–**I**) Mid pachytene: lack of interstitial asynapsis, some of the SC bivalents with short asynaptic forks, and some of the SC bivalents without asynaptic forks. (**J**–**L**) Early diplotene: segment-by-segment SYCP3 elimination from lateral elements, desynaptic axes and telomeric forks. Other images of this nucleus are shown in Figures S1G,G1 and S7G–I. Parts of the images of the nuclei (yellow outline in **A**,**D**,**G**,**J**) are enlarged (**B**,**E**,**H**,**K**). Schemes of the configurations of the axial elements and lateral elements of the SC are presented in (**C**,**F**,**I**,**L**). Scale bar = 5 µm.

**Figure 3:**
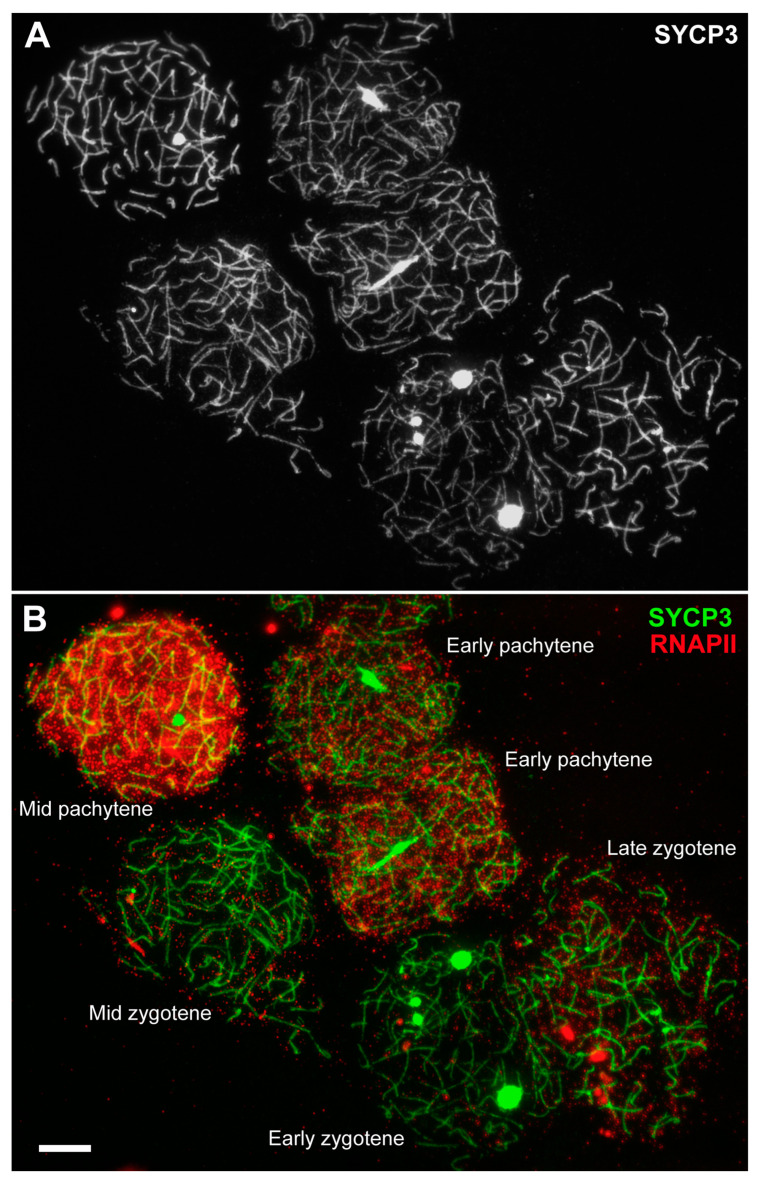
Differential distribution of the RNA polymerase II (RNAPII) signals through lamprey prophase I. Images of SYCP3 (gray) (**A**), SYCP3 (green) and RNAPII (red) proteins (**B**) in spermatocytes. Scale bars = 5 µm.

To analyze the structure and behavior of meiotic chromosomes, immunodetection of the SYCP3 protein (a major protein of axial/lateral elements of the SC) was performed. Different anti-SYCP3 antibodies were used as follows: mouse monoclonal antibodies (Abcam, cat. #97672, #205846) were raised against the mouse protein, and a rabbit polyclonal antibody (Abcam, cat. #15093) was raised against the C-terminus of the human protein. Only the latter yielded a positive specific signal. In leptotene spermatocytes, the SYCP3 signal was found to be diffusely distributed throughout the nucleus with SYCP3-positive DAPI-negative round-like bodies of various sizes, which are conventionally referred to as RLBs (round-like bodies; Figure S1A,A1,B,B1). Such a tentative name was given due to a lack of information regarding their true nature. RLBs were not detected in the positive and negative controls (Figures S2 and S3). We employed antibodies to detect the location of fibrillarin—an element of the nucleolar dense fibrillar component—but no specific signals were found. At the end of leptotene, forming SYCP3 axes were formed (Figure S1B,B1). In some axes and SCs, round or oval SYCP3- and DAPI-positive fibrillarin-negative structures were visible at all substages, which were provisionally named SC-associated structures (SCASs; Figures S1B,B1,C,C1,E,E1,F,F1,G,G1, S4 and S5). The chromatin around this structure (on the periphery) stained more intensely than that in the central part (Figure S1C,E,G). The variation in the number of RLBs was wide, ranging from 0 (e.g., Figure 1G) to over 60 (Table S1). Thin SYCP3 axes and short fragments of SCs were visible in the early zygotene (Figure 1A–C and Figure S6A). In mid-zygotene, the SYCP3 axes gave rise to short and long SC fragments (Figure 1D–I and Figure S1C,C1,D,D1). By the end of zygotene, most of the SC bivalents were formed, although they did so with incomplete synapsis in the terminal chromosome regions (which we referred to as asynaptic forks) and in some areas of interstitial and distal asynapsis for some SCs (Figure 1J–L, Figures S1E,E1,E2,E3 and S4A,B). In early pachytene, SCs did not have interstitial and distal asynapsis, and most SC bivalents had asynaptic forks of different lengths and widths (Figure 2A–C and Figure 3, Figure S6D). In mid-pachytene, some of the SCs had asynaptic forks, and some of the SCs did not (Figure 2D–I, Figures S1F,F1,F2,F3 and S4C). At the beginning of diplotene (Figure 2J–L), chromosomal forks were still present (Figure S1G,G1,G2,G3); therefore, it is likely that some of the bivalents undergo incomplete synapsis throughout the first prophase. Chromosome desynapsis was terminal for some chromosomes and interstitial for some others (Figure S1G2,G3). The elimination of the SYCP3 protein from the desynaptic axes proceeded segmentally (as if disappearing in small parts from the axes, alternating SYCP3-positive and SYCP3-negative sites; Figure S1G2,G3). Pachytene and diplotene showed one bivalent each, with an SCAS in the central part of the nucleus (Figure S1F,F1,G,G1). Both SCASs and RLBs were AgNO3-positive (Figure S5).

**Figure 4:**
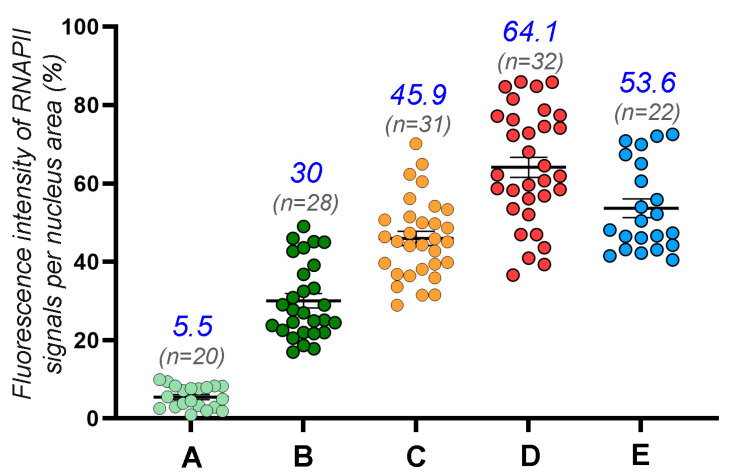
Quantitative data on the distribution of the RNAPII signals at different stages of prophase I. Scatterplot depicts the fluorescence intensity of the RNAPII signals per meiotic nucleus area (%). The scatterplot is based on data from Table S2. (**A**) Mid zygotene; (**B**) Late zygotene; (**C**) Early pachytene; (**D**) Mid pachytene; (**E**) Early diplotene. All dot columns are significantly different from each other.

In prophase I, various configurations of axial elements and SCs were observed. Stand-alone univalents were seen in early and mid pachytene (Figure S1E1,F1). In the single bivalents, lateral elements of different lengths were noted (Figure S4B2,B3 and Figure 4). The axial elements in the asynaptic forks of two different bivalents could be located very close to each other, resembling a trivalent configuration (Figure S4B,C), similar to the bicentric trivalent SC configurations previously found in sturgeons (*Acipenser transmontanus*) [35]. It is difficult to say whether such configurations are an artifact or true compounds, but it is necessary to pay attention to them.

To identify centromeric chromosome regions, antibodies to kinetochore proteins, human CREST, and ACA were used. These antibodies did not give a positive specific signal under different incubation conditions.

### 3.2. Transcription in the First Meiotic Prophase in the Lamprey

The assay of transcriptional activity was carried out by the simultaneous immunostaining of the SYCP3 protein and phosphorylated RNA polymerase II (RNAPII-CTD-Phospho-Ser5, abbreviated as RNAPII). Differential distributions of the signal throughout prophase I were documented. There was no RNAPII signal in the leptotene. In early and mid zygotene, a weak RNAPII signal arose as stand-alone dots of various sizes (Figure 3, Figures S6A,B and S7A–C), comprising 5.5% of the signal from the nucleus area (Figure 4, Table S2). In late zygotene, the RNAPII signal increased up to 30% (Figure 3, Figure 4 and Figure S6C, Table S2). In early pachytene, the RNAPII signal increased to 46% (Figure 3, Figure 4 and Figure S6D, Table S2), and it became denser and brighter by mid pachytene (65%; Figure 4, Figures S6F and S7D–F) and early diplotene (53.6%; Figure 4 and Figure S7G–I, Table S2). The RNAPII signal was not specifically associated with unpaired and paired segments, including asynaptic forks (Figure S6D,E). That is, there was no regular variation in RNAPII points along and around the bivalents with completed synapsis or those with bivalents that were not fully synapsed (Figure S6D1–D10,E3,E4,H). Thus, the initiation of transcriptional reactivation is not associated with the completion of chromosomal synapsis (Figure S6H). In diplotene, the RNAPII signal has a pattern similar to that in mid pachytene (Figures S6F,G and S7D–I). The RNAPII signal is diffusely scattered as dot foci throughout meiotic nuclei (Figure S6D–G), with occasional RNAPII signal clumps that are denser (Figures S6D,G and S7). SCASs tend to have fewer weak RNAPII foci (Figure S6E1,E2,G1,G2) or no RNAPII signal at all (Figure S6D,D10,F1,F2), likely indicating either low transcriptional activity or its absence in these structures.

**Figure 5:**
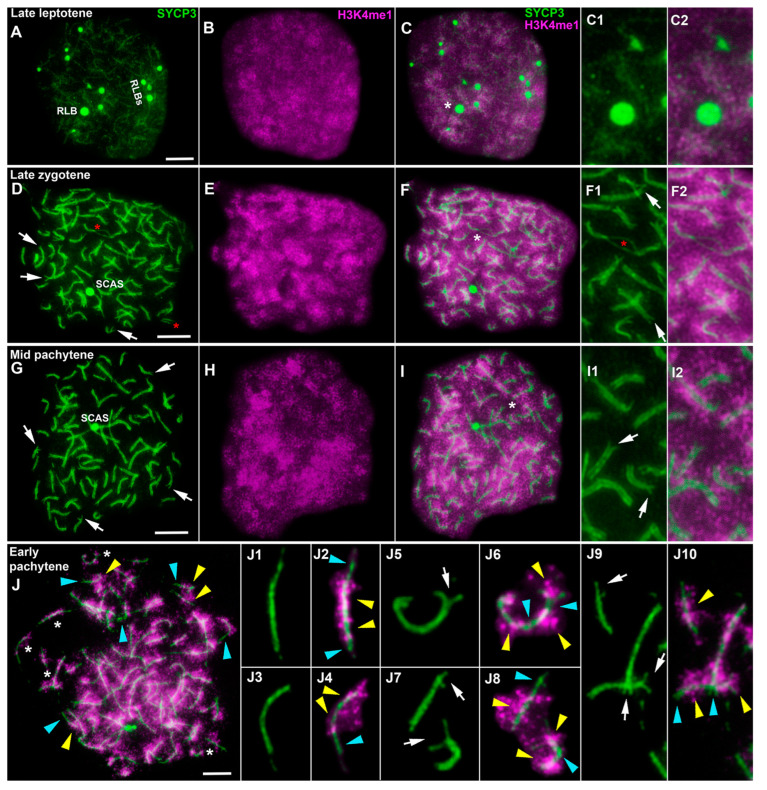
Immunolocalization of SYCP3 (green) and H3K4me1 (magenta) proteins in lamprey spermatocytes at different stages of prophase I. White arrows indicate asynaptic forks in telomeric regions of SC bivalents. Yellow arrowheads point to H3K4me1-positive (enriched) regions of the SCs. Blue arrowheads indicate H3K4me1-negative (not enriched) regions of the SCs. Enlarged regions of nuclei are shown as white asterisks. SCAS: an SC-associated structure in a long SC. RLB: a round-like body. (**A**–**C**) Late leptotene. Thin, weak SYCP3 axes and SYCP3 lumps of various shapes and sizes are visible. The H3K4me1 signal is evenly distributed throughout the nucleus. The H3K4me1 signal is more monomorphically distributed compared to subsequent stages (**C1**,**C2**). RLBs are noticeable in the nucleus. (**D**–**F**) Late zygotene. Zones of interstitial asynapsis in some bivalents or short univalents (red asterisks) are seen (**D**,**F1**,**F2**). RLBs are absent. The H3K4me1 signal is distributed across the nucleus, but distinct domains with a brighter H3K4me1 signal (**E**) are present. (**G**–**I**). Mid pachytene. Asynaptic terminal sites are retained in many bivalents (**G**,**I1**). Both H3K4me1-positive and H3K4me1-negative domains (**H**,**I**,**I1**,**I2**) are observed in the nucleus. An SCAS is localized to one of the longest SCs (**G**,**J**). A well-spread nucleus at the stage of early-mid-pachytene. The H3K4me1 signal is distributed within chromatin around SC bivalents. In the SC bivalents, areas with and without a bright H3K4me1 signal (**J1**–**J10**) are visible. Scale bars = 5 µm (**A**–**J**).

Immunocytochemical examination of the distribution of other proteins related to transcriptional regulation failed to detect specific signals of mouse antibodies against γH2AX and H3K9me3. A more detailed selection of immunostaining conditions can perhaps help to determine specific signals. Species specificity of the antibodies may be the reason for the lack of immunostaining specificity. Furthermore, the choice of new antibodies, as well as new methodological conditions and approaches, may help to determine the localization of these proteins in lamprey meiocytes. It is believed that histone H3K4me1 is associated with the transcriptional repression of chromatin [36]. Double immunolabeling with antibodies against SYCP3 and H3K4me1 yielded specific staining. Several weak SYCP3 signals in the form of lumps were visible in preleptotene, and the H3K4me1 signal was very weak (slightly above the background level; Figure S8). By late leptotene to early zygotene, the amount of the H3K4me1 signal had sharply increased (Figure 5A–C and Figure S8). In mid zygotene, the H3K4me1 signal was bright (Figures S8–S10). In leptotene and zygotene, the H3K4me1 signal proved to be monomorphically and diffusely distributed throughout the nucleus (Figures S8 and S10). From the middle and/or end of zygotene to mid pachytene, the H3K4me1 signal looked like separate regions that were either more or less intense. The more intense H3K4me1 domains were located around an SC, while the less bright ones were situated between SCs (Figure 5D–F, Figures S9 and S10). This was especially noticeable in well-spread nuclei. Some bivalent segments were both H3K4me1-positive and H3K4me1-negative (Figure 5J). At first, we assumed that this phenomenon was due to the degree of the completion of synapsis because the intensity of the H3K4me1 signal was slightly lower in asynaptic areas (Figure 5F1,F2). Nonetheless, this signal varied between SC bivalents with complete synapsis and SC bivalents with asynaptic forks (Figure 5J1–J10). Asynaptic forks were either H3K4me1-positive (Figure 5J5,J6) or H3K4me1-negative (Figure 5J7–J10). The number of diplotene nuclei was extremely small. A combination of features such as stand-alone SYCP3 axes, the absence of RLBs and bouquet-like configurations, and a domain-like distribution of H3K4me1, helped us to assign the nuclei to the diplotene stage (Figure S7G–I).

## 4. Discussion

**Figure 6:**
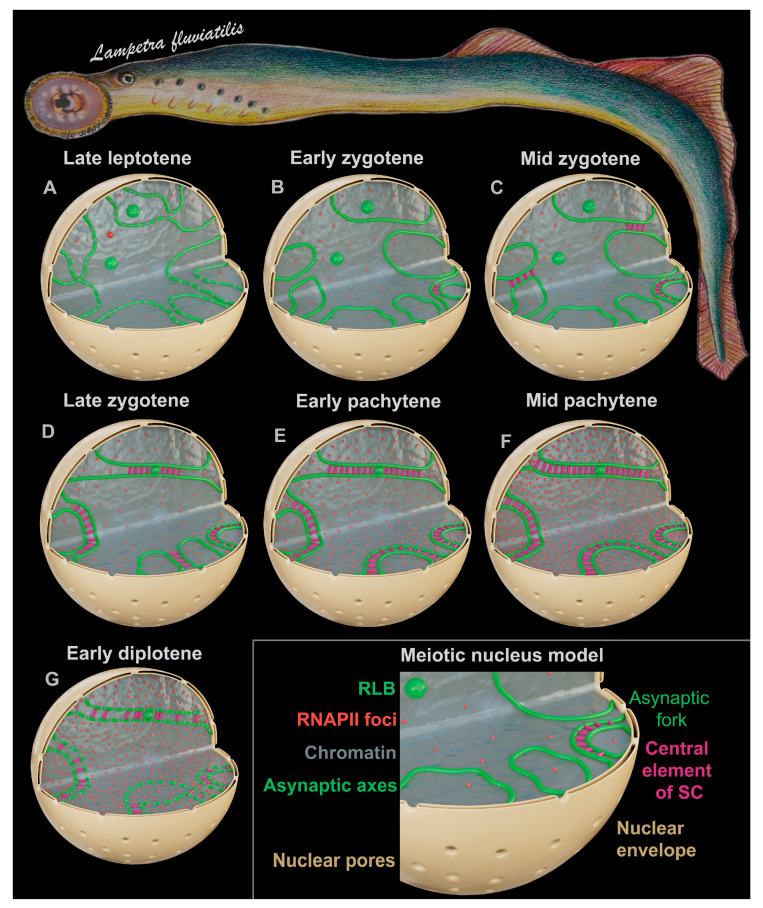
Simulation of meiotic nuclei at different substages of the first prophase in lamprey *Lampetra fluviatilis*. This scheme reflects the features of chromosome synapsis and transcription and does not reflect recombination and chromatin landscape. Schematic representations of (1) SYCP3-positive axial elements and lateral elements of SCs are shown with green lines/axes, (2) central elements of SCs are shown with short magenta lines between green lines, (3) RNA polymerase II (RNAPII) foci (transcriptional marker) are shown red dots, (4) chromatin is shown gray color, (5) asynaptic forks are shown in the telomeric regions of the SC bivalents, (6) round-like bodies (RLBs) and SC-associated rounded structures (SCASs) are shown as green balls, (7) the nuclear envelope and its pores are shown in sand color. (**A**) Late leptotene. Thin axial elements (axes) with segments of different brightness are formed. RNAPII is visualized at a low level (rare red globules and dots). (**B**) Early zygotene. The axes begin to approach each other, and some of them begin to synapse (a fragment of the SC is formed in the nucleus, and there is a central element). RNAPII level is slightly higher compared to the previous stage. (**C**) Mid zygotene. Numerous SC fragments are formed between the axes. The RNAPII level is approximately the same as the previous stage. (**D**) Late zygotene. Most of the SCs are formed, although they do so with incomplete synapsis in the terminal chromosome regions (which we refer to as asynaptic forks) and in some areas of interstitial and distal asynapsis for some SCs. RNAPII level is two to three times higher than the previous stage. (**E**) Early pachytene. SCs do not have interstitial and distal asynapsis, and most SC bivalents have asynaptic forks of different lengths and widths. RNAPII level is higher compared to the previous stage. (**F**) Mid pachytene. Some of the SCs have asynaptic forks, and some of the SCs do not. RNAPII level is maximum (the number and density of red dots are the highest). (**G**) Early diplotene. Desynapsis is terminal for some chromosomes and interstitial for some others. The elimination of the SYCP3 protein from the desynaptic axes proceeds segmentally (green lines are intermittent). RNAPII level is at the same or slightly lower than in the previous stage.

It would seem that extensive studies on classic experimental models lead to coherent ideas about various biological processes. By contrast, research on non-model and taxonomically distant objects bring a smorgasbord, if not some chaos, into the picture. In the context of chromosomal and meiotic studies, the European river lamprey is a non-conventional species that has already helped to clarify the details of an interesting process, namely genome elimination. It has been proven that a large part of the genome and even entire chromosomes that are specific to germ cells are lost during the early stages of embryonic development in the sea lamprey *Petromyzon marinus* [26,27]. In the present work, the analysis of chromosome synapsis and of the transcriptional landscape of prophase I chromatin in the lamprey enabled us to add some new data to the existing concepts of synapsis and transcription. We summarize the data in the form of simulations of meiotic nuclei at different substages of prophase I (Figure 6).

### 4.1. Not All Chromosomes Show Completed Synapsis

Few papers are devoted to chromosomes during lamprey meiosis. Robinson and Potter were the first to report on chromosomes during meiosis I in *Mordacia praecox* [37] and *Geotria australis* [38]. From then onwards, meiotic studies have contributed to the investigation of the phenomenon of genome elimination in sea lampreys [27,39]. Until now, however, chromosome synapsis and the processes that take place in chromatin have not been the focus of attention. 

The analysis in the present study of chromosome synapsis in the river lamprey here made it possible to reveal a number of unusual features (Figure 6 and Figure S11). Firstly, we were unable to find a single pachytene nucleus where all chromosomes showed completed synapsis (instead, there were 6–10 chromosomes without full synapsis). It remains to be clarified whether this is a species-specific feature or the stage of completed synapsis is still in progress remains to be clarified. We can theorize that such a stage may be very brief; accordingly, we were unable to identify it. Be that as it may, a very similar case has been observed in the sturgeon *A. transmontanus* [35]. Although the authors did not emphasize this phenomenon, none of the presented spreads contained chromosomes with fully completed synapsis (asynaptic forks were present). It seems that the presence of asynaptic regions at pachytene should lead to pachytene arrest [40,41,42,43]. This pattern was discovered in mammals but has not been experimentally confirmed in other vertebrate taxa. In chickens with chromosomal asynapsis due to heterozygosity for translocations, no impairment of spermatogenesis has been detected [44]. Notably, triploid Darevskia lizards with pronounced chromosome asynapsis form spermatozoa, but the vast majority of them have morphological aberrations [45]. The latter observation confirms the notion that the absence of a pachytene checkpoint may underlie the formation of polyploid species [46]. This notion may explain, to some extent, the terminal segments of asynapsis in paleotetraploid sturgeon [35]. It is likely that the presence of asynaptic forks in lamprey SC bivalents does not prevent further meiocyte progression, which may be due to the absence of the pachytene checkpoint or its nonstandard functioning. 

Secondly, using immunocytochemical methods and electron microscopy, trivalent-like SC configurations were found in the lamprey. Although we are still unsure about the truth of trivalent-like configurations, if they exist, then this combines them with the sturgeon [35]. The authors call them bicentric gap configurations and believe that such nuclei can be aneuploid. The similarity in behavior between lamprey and sturgeon meiotic chromosomes seems to be no accident because they share a high diploid number (with macro- and minichromosomes). It cannot be ruled out that the possible paleoploid origin of the karyotype of both species may contribute to such synaptic patterns.

Thirdly, we documented the presence of special bodies called RLBs and SCASs. The first idea that comes to mind is that RLBs may be a morphological manifestation of hypothetical chromatin elimination because outwardly similar micronuclei have been identified during early embryogenesis in the sea lamprey [47]. Nevertheless, these meiotic bodies were DAPI-negative and, therefore, unlikely to be related to chromatin. Additionally, they probably do not have a nucleolar nature (negative fibrillarin immunostaining). Most likely, these structures are transcriptionally inactive because only rare dim dot-like “transcriptional” signals were present in them, just as within SCASs. An SCAS emits a more intense DAPI signal at the periphery than in the center. At the same time, both types of bodies proved to be subject to silver staining. Thus, the events and structures in prophase I of the river lamprey both share some similarities with sturgeon and possess a series of unique features.

### 4.2. Lamprey Prophase I Is Characterized by Some Unique Epigenetic Patterns

The remodeling and modification of histones during meiosis are processes of a complex epigenetic regulatory system that determines the active or silent state of chromatin. It has been assumed that asynaptic chromatin is subject to transcriptional silencing [15], and the completion of chromosomal synapsis and the repair of DNA double-strand breaks lead to the resumption of transcription, which is reflected in the “synapsis or silencing” model [48]. Later, it was found that a more complex epigenetic program exists in prophase I because transcriptional reactivation does not begin immediately after the completion of synapsis [12]. To determine the activity of transcription at different stages of lamprey prophase I, we tested RNAPII activity. This type of polymerase is the central coordinator of the transcription machinery. The phosphorylation of the C-terminal domain (CTD) of RNAPII regulates and coordinates transcription and chromatin remodeling and modifications [49,50,51]. We used antibodies to the RNAPII CTD phosphorylated at serine 5 in tandem repeated heptapeptides YSPTSPS. In the lamprey, during the late leptotene and early zygotene, the first transcriptional signals appear in the form of rare RNAPII globules and dots when the chromosomes have not yet entered synapsis. In the mid zygotene, a greater amount of RNAPII signals appear. RNAPII can likely already be synthesized in the leptotene and early zygotene, but at an extremely low level. It is possible that synapsis itself initiates transcriptional reactivation at mid zygotene. Most likely, transcriptional reactivation begins at the transition from the early to the mid zygotene and increases sharply towards the beginning of the pachytene. This transcription pattern differs from that previously identified in mammals, where reactivation starts in mid pachytene [12,13,52,53]. We propose that transcriptional reactivation in lamprey prophase I is not connected with the completion of chromosome synapsis. The same conclusion was made earlier in a meiotic study on true bugs [54]. The authors believe that the absence of some repressive chromatin modifications in early prophase I may be responsible for the earlier reactivation. Given that we failed to detect γH2AFX and H3K9me3, we can assume that this hypothesis is applicable to the lamprey. On the other hand, the absence of transcription in leptotene and early zygotene must be explained by some repressive chromatin mechanisms. It is likely that there are some other histone modifications or processes that ensure the silent state of chromatin. This topic requires further research.

Our results on the H3K4me1 histone distribution in lamprey prophase I are no less intriguing. From leptotene to diplotene (inclusively), an intense H3K4me1 signal was observed throughout the meiotic nucleus with one specific feature: the further the progression of prophase I, the more clustered the histone localization was. This distribution differs from what has previously been registered in wild mice [12,13,19]. Because the amount of H3K4me1 diminishes in mid pachytene during mouse meiosis, it has been proposed that this histone is related to silencing [12], especially because a more aberrant H3K4me1 pattern is seen in the sex body of mutant meiocytes [19]. In the case of the lamprey, however, during the transition to the active transcriptional state of chromatin in mid-to-late zygotene, the amount of H3K4me1 stayed unchanged (did not decrease or increase). These findings suggest that H3K4me1 is probably not involved in the cycle of inactivation–reactivation of transcription. Notably, recent research on murine and human germ cells suggests that H3K4me1 may help to implement a transcriptionally neutral gene state that could be activated [55]. It has also been reported that the H3K4me1 histone in somatic cells in the absence of the trimethylated form of H3K4 may correlate with gene repression; on the other hand, this histone has been detected in active gene promoters flanking H3K4me3 [36]. According to the reasoning of Bae and Lesch [55], H3K4me1 may play a context-dependent role in transcriptional regulation.

The function of the H3K4me1 histone in lamprey prophase I remains unknown. Moreover, in well-spread meiocytes, H3K4me1 was found to be differentially distributed along the SC, suggesting that it can label regions related to different chromatin conformations. High-resolution microscopy has enabled some authors to identify distinct actively transcribed (H3K4me3-positive, loop-like) and repressed (H3K27me3-positive) clusters along pachytene chromosomes [56]. The alternation of H3K4me1-positive and -negative loop-like clusters in lamprey meiotic chromosomes (regardless of synapsis completion) may be due to some kind of chromatin state (possibly transcriptionally neutral) that is not directly connected to transcription. However, it definitely does not prevent this process, nor does it preclude repression in early prophase I. A clear picture of the functions of H3K4me1 in meiotic chromatin is gradually coming into focus.

## 5. Conclusions

Research on the epigenetics of meiosis is still at an active stage of research, and the discovery of new data helps researchers draw nearer to a more complete picture of the functioning of various processes, such as transcription, silencing, and chromatin modification/remodeling. Here, in the European river lamprey, we identified not only unusual synaptic patterns and the absence of a connection between the completion of synapsis and the onset of reactivation but also a unique pattern of the distribution of monomethylated histone H3K4. This pattern has yet to be interpreted, which indicates that many details of meiotic chromatin epigenetics remain unknown. The question of where some of the identified differences from the published data come from is tricky because currently, the data for a large-scale assessment of the functioning of the lampreys’ meiotic chromatin are currently deficient. Nonetheless, we should not forget that the jawless taxa split off from the main branch of the vertebrate evolutionary tree (jawed animals) approximately 535–462 million years ago [22,23,24], and considerable evolutionary shifts/modifications of biological processes may have occurred during this long period. Additionally, the identified specific features may be a part of the wide variation in epigenetic patterns. Further research will help to clarify and decipher points of difference and similarity. Lamprey meiosis represents a new prism through which many more discoveries may be detected.

## Data Availability

Not applicable.

## Supplementary Materials

The following supporting information can be downloaded at: https://www.mdpi.com/article/10.3390/life13020501/s1, Table S1: The average number of round-like bodies (RLBs) per nucleus in lamprey spermatocytes at different substages of prophase I; Figure S1: Chromosome synapsis at prophase I in the lamprey; Figure S2: Positive control of immunostaining of lamprey spermatocytes; Figure S3: Negative control of immunostaining of lamprey spermatocytes; Figure S4: Chromosome synapsis in lamprey spermatocytes at late zygotene to mid pachytene; Figure S5: Electron microscopic microphotos of lamprey spermatocytes at the pachytene stage; Figure S6: Immunolocalization of the SYCP3 protein and RNA polymerase II (RNAPII) in lamprey spermatocytes at different stages of prophase I; Figure S7: Immunolocalization of SYCP3 and RNA polymerase (RNAPII) proteins in lamprey spermatocytes at the prophase I; Table S2: The average mean of fluorescence intensity of RNAPII signals per nucleus area (%) in lamprey spermatocytes at different substages of prophase I; Figure S8: Immunolocalization of SYCP3 and H3K4me1 proteins in lamprey spermatocytes at different stages of prophase I; Figure S9: Immunolocalization of SYCP3 and H3K4me1 proteins in lamprey spermatocytes at different stages of prophase I; Figure S10: Immunolocalization of SYCP3 (green) and H3K4me1 (magenta) proteins in lamprey spermatocytes at different stages of prophase I; Figure S11: The meiotic first prophase in European river lamprey *Lampetra fluviatilis*.
