# Supplementary material for: The First Analysis of Synaptonemal Complexes in Jawless Vertebrates: Chromosome Synapsis and Transcription Reactivation at Meiotic Prophase I in the Lamprey Lampetra fluviatilis (Petromyzontiformes, Cyclostomata)
Source: Life (Basel). 2023 Feb 11;13(2):501. doi: 10.3390/life13020501 (PMC9959970; doi:10.3390/life13020501)
Supplement: Supplementary file 1 [file life-13-00501-s001.zip › life-2153313-supplementary.pdf]

life

MDPI

Supplementary material

The First Analysis of Synaptonemal Complexes in Jawless Vertebrates: Chromosome Synapsis and Transcription Reactivation at Meiotic Prophase I in the Lamprey *Lampetra fluviatilis* (Petromyzontiformes, Cyclostomata)

Sergey Matveevsky [1,*], Nikolay Tropin [2], Aleksandr Kucheryavyy [3] and Oxana Kolomiets [1]

[1] Vavilov Institute of General Genetics, Russian Academy of Sciences, 119991 Moscow, Russia; olkolomiets@mail.ru
[2] Vologda Branch of the Russian Federal Research Institute of Fisheries and Oceanography, 160012 Vologda, Russia; nikolay-tropin1@yandex.ru
[3] Institute of Ecology and Evolution, Russian Academy of Sciences, 119071 Moscow, Russia; scolopendra@bk.ru
* Correspondence: sergey8585@mail.ru

Table S1. The average number of round-like bodies (RLBs) per nucleus in lamprey spermatocytes at different substages of prophase I

		1	2	3
	Lamprey	Zygotene, M, (*n* nuclei), [range]	Pachytene, M, (*n* nuclei), [range]	Diplotene, M, (*n* nuclei)
a	LF-01	29.10, (*n*=10), [1-62]	1.61, (*n*=13), [0-9]	0, (*n*=6)
b	LF-02	17.55, (*n*=11), [0-51]	1.00, (*n*=13), [0-6]	0, (*n*=5)
c	LF-03	9.48, (*n*=29), [0-43]	0.38, (*n*=70), [0-7]	0, (*n*=8)
	Mean (M±SD)	**18.71**±9.8	**0.99**±0.61	**0**
	Significant (P < 0.05)	1a/1c	2a/2c	
	Not significant	1a/1b, 1b/1c	2a/2b, 2b/2c	

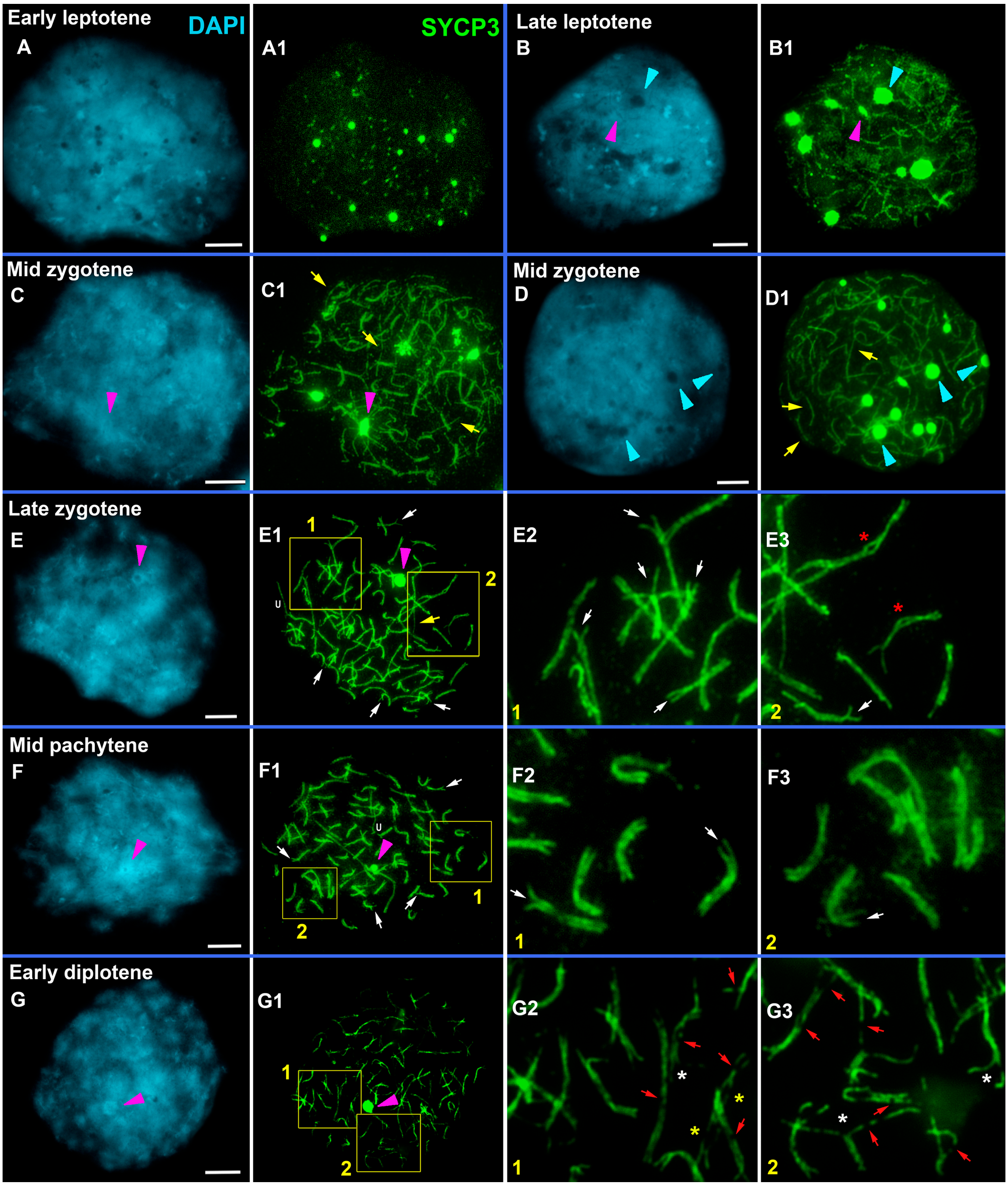

Figure S1. Chromosome synapsis at prophase I in the lamprey. SYCP3 staining (green) revealed the structure and behavior of axial and lateral elements of an SC. DAPI (cyan) stains chromatin. White arrows indicate asynaptic forks in telomeric regions of SC bivalents. Yellow arrows indicate asynaptic axes. Blue arrowheads point to DAPI-negative rounded bodies (RLBs). Enlarged areas of nuclei are shown in yellow numbers. Pink arrowheads indicate SC-associated rounded structures (SCASs). "U" stands for univalents. **A,A1.** Early leptotene. A diffuse SYCP3 signal is distributed throughout the nucleus. Numerous DAPI-negative RLBs of various sizes are seen. **B,B1.** Late leptotene. SYCP3 fragments and axes appear. The diffuse SYCP3 signal is still present throughout the nucleus. Both RLBs and SCASs are present in the nucleus. **C,C1.** Mid zygotene (see Fig. S6A–C). Chromosomal axes enter synapsis, forming short and long SC fragments. There is one SCAS in this cell. **D,1** Mid zygotene. A large number of axes are unsynapsed. Numerous RLBs and SCASs are visible. **E,E1.** Late zygotene. Some bivalents retain short asynaptic interstitial regions (red asterisks). Most bivalents have terminal regions of incomplete synapsis (asynaptic "forks") (**E2**, **E3**). One of the long SCs has an SCAS in the central part. **F,F1.** Mid pachytene. Some of the bivalents show complete synapsis, although most of the SCs retain asynaptic "forks" (**F2**, **F3**). One of long SCs contains an SCAS in the central part. **G,G1.** Early diplotene (see Fig. S6G–I). Desynapsis is accompanied by segment-by-segment removal of the SYCP3 protein from the axes (red arrows). Desynapsis can start from both terminal (white stars) and interstitial (yellow stars) regions of chromosomes (**G2**, **G3**). Scale bar = 5 μm.

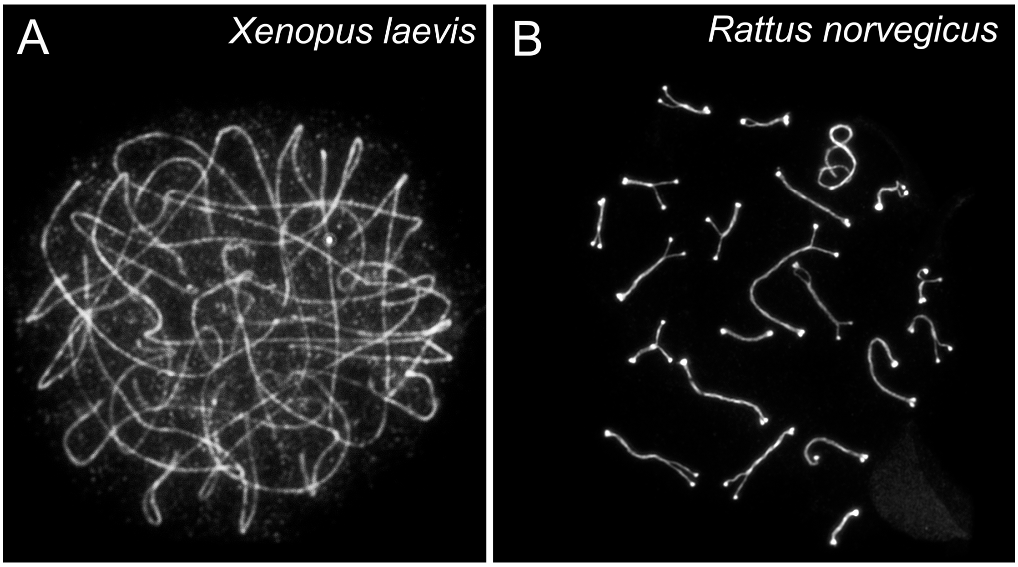

Figure S2. Positive control of immunostaining of lamprey spermatocytes. Staining with the same primary (SYCP3) and secondary (goat anti-rabbit Alexa Fluore 488) antibodies for frog (A) and rat (B) spermatocytes. RLBs have not been identified.

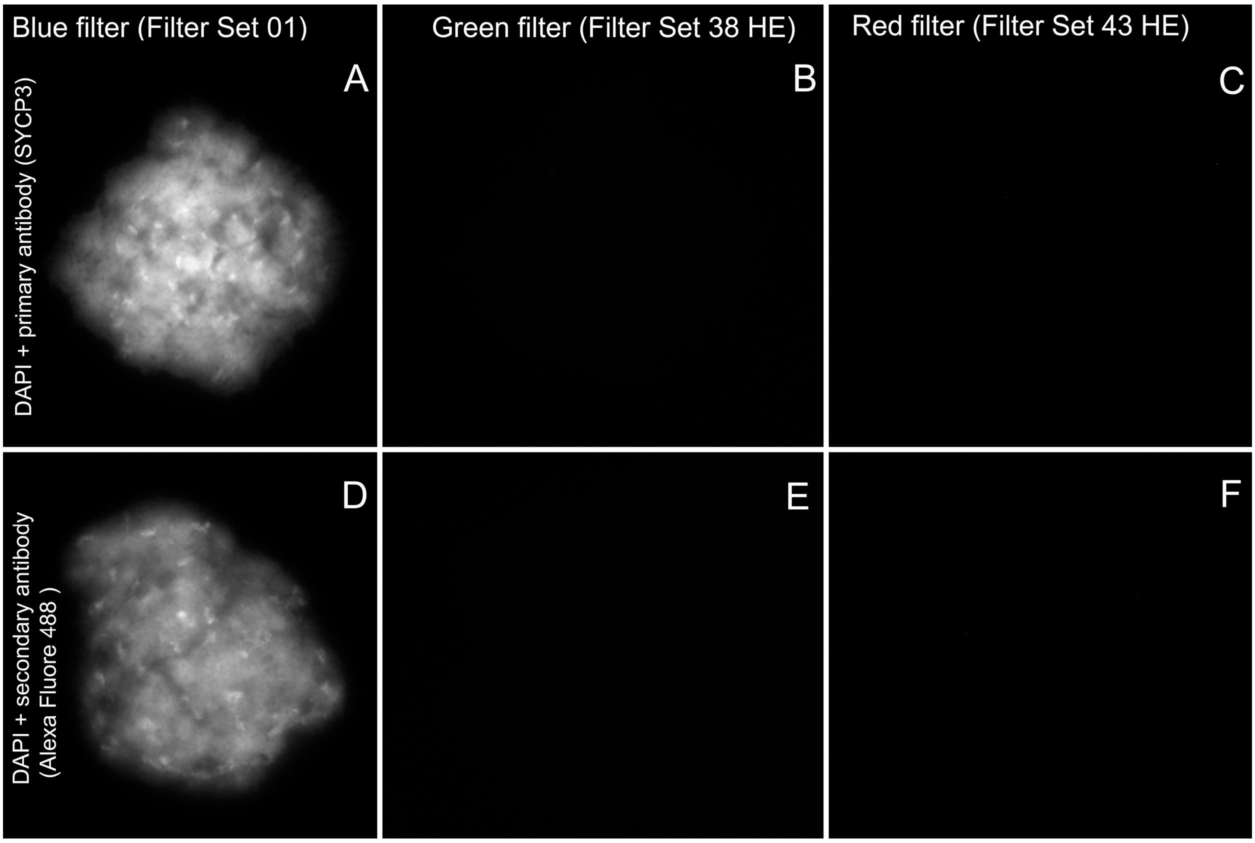

Figure S3. Negative control of immunostaining of lamprey spermatocytes: **A-C.** Overnight incubation with primary antibody (SYCP3); **D-F.** Overnight incubation with secondary goat anti-rabbit Alexa Fluore 488 conjugated antibodies. To identify nuclei, the slides were stained with DAPI (Filter set 01, left column). At standard exposure, there were no immuno-signals on the green (Filter set 38HE, central column) and red (Filter set 43HE, right column) CHROMA filters.

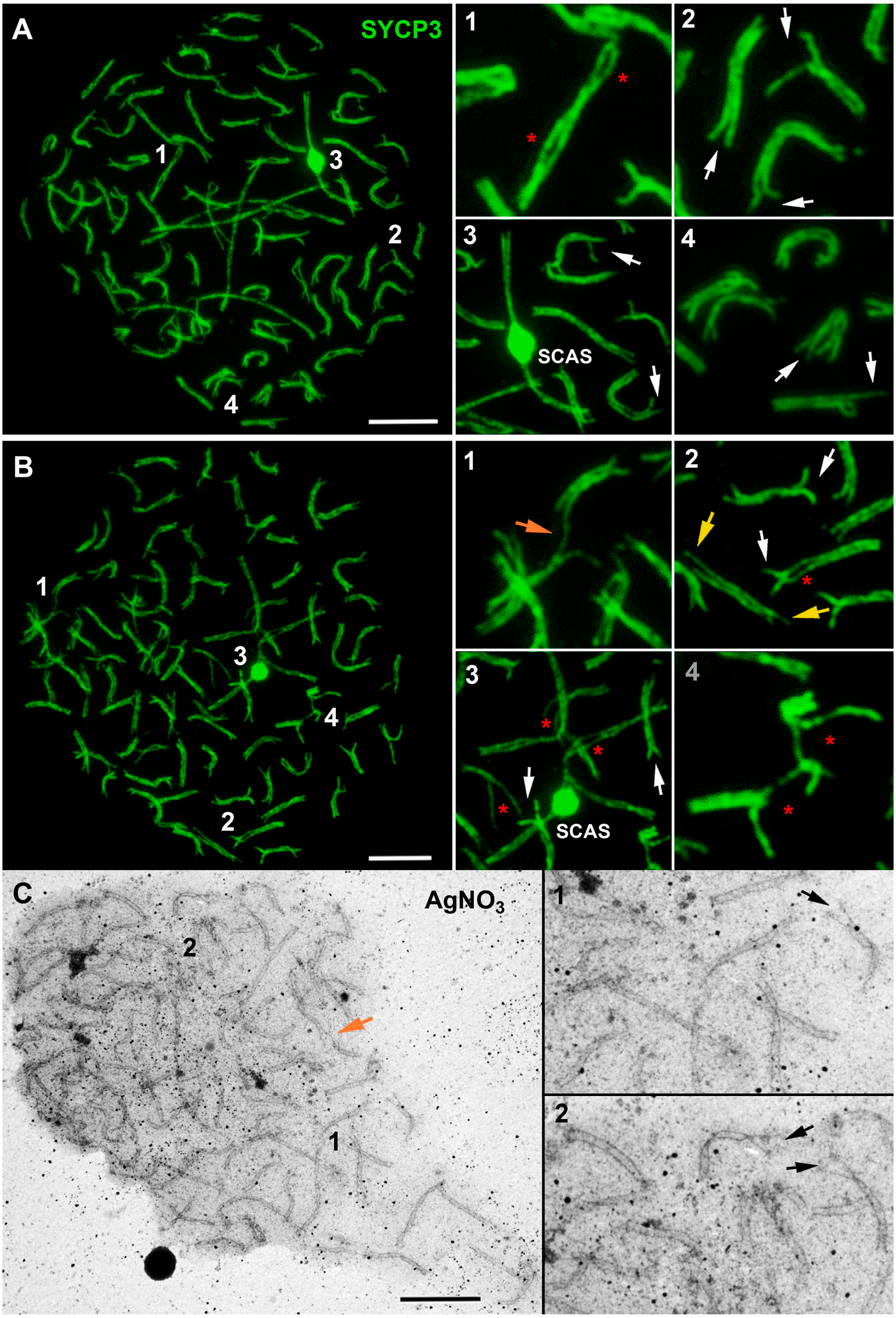

Figure S4. Chromosome synapsis in lamprey spermatocytes at late zygotene (**A, B**: light microscopy) to mid pachytene (**C**: electron microscopy). SYCP3 staining (green) revealed the structure and behavior of the axial and lateral elements of an SCs. White arrows (for A2–A4 and B2–B4) and black arrows (for C1 and C2) indicate asynaptic forks in telomeric regions of SC bivalents. The orange arrow points to the connection of axial elements of two bivalents, forming a trivalent-like SC configuration. The numbers indicate enlarged parts of the nucleus shown on the right. SCAS: an SC-associated structure in large SCs. Red asterisks indicate interstitial asynaptic sites. Yellow arrows point to different lengths of two axial/lateral elements in one bivalent. Scale bar = 5 μm.

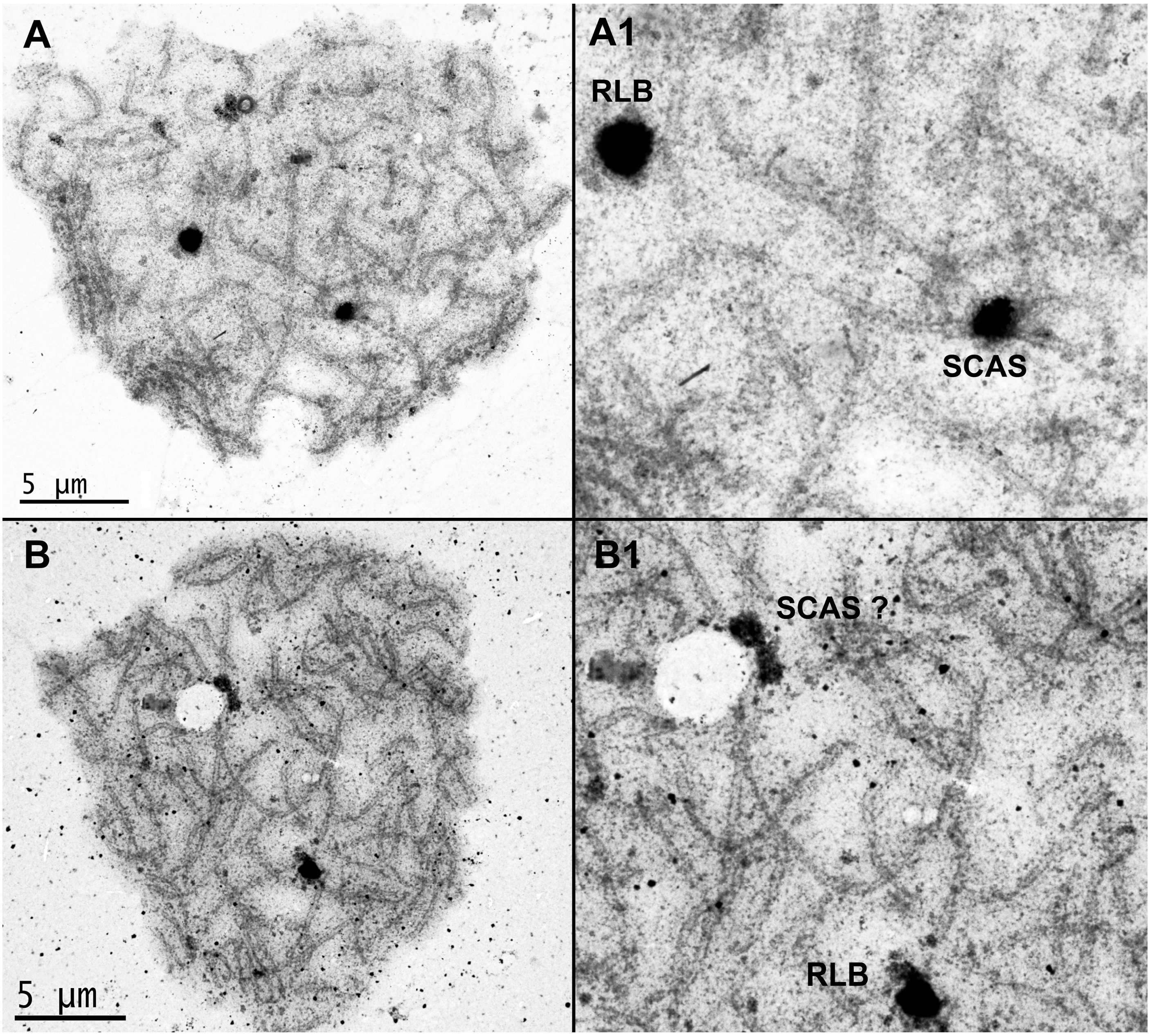

Figure S5. Electron microscopic microphotos of lamprey spermatocytes at the pachytene stage (**A**, **B**). SCAS: an SC-associated structure in large SCs. RLB – round-like body. Enlarged areas of nuclei are shown in **A1**, **B1**.

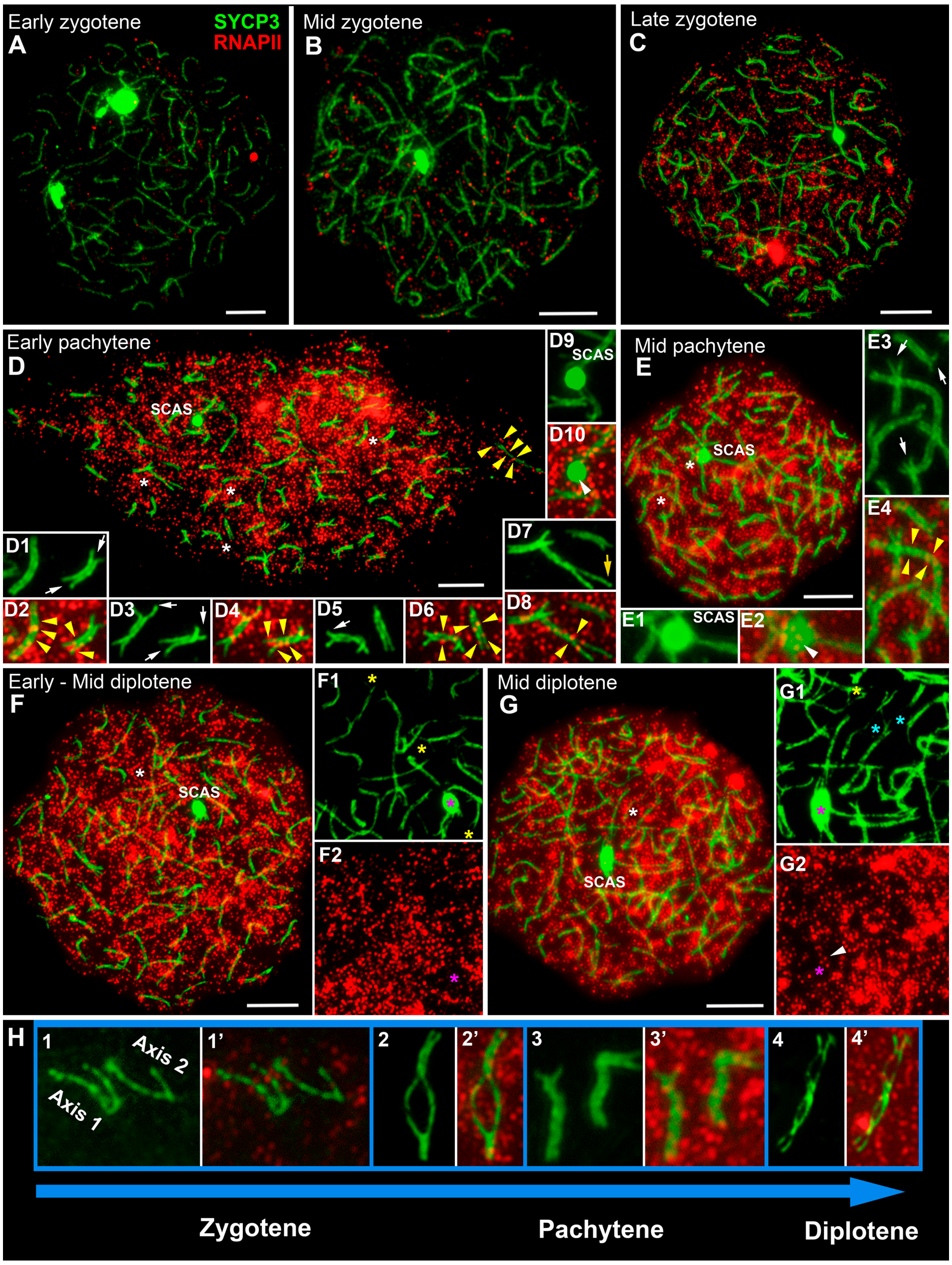

Figure S6. Immunolocalization of the SYCP3 protein (green) and RNA polymerase II (RNAPII, red) in lamprey spermatocytes at different stages of prophase I. White arrows indicate asynaptic forks in terminal regions of bivalents. Yellow arrowheads point to variable localization of RNAPII foci along the synaptic and asynaptic segments of the SC bivalents. SCAS: an SC-associated structure in a long SC. White arrowheads indicate rare stand-alone RNAPII signals within a SCAS. Enlarged regions of nuclei are shown as white asterisks. **A.** Early zygotene. Thin weak SYCP3 axes and SYCP3 lumps of various shapes and sizes are visible. The RNAPII signal is in the form of rare small dots. **B.** Mid zygotene. A large number of RLBs arose throughout nuclear chromatin. The number of rare RNAPII foci of different sizes is greater than that in the previous substage. **C.** Late zygotene to early pachytene. The number of RLBs is less than that in the previous stage. Numerous RNAPII dots, weak in intensity, emerge throughout the nucleus. **D.** Transition from early to mid pachytene. Most of SCs carry terminal asynaptic forks. An intense dot-like pattern of RNAPII signals is seen in the nucleus. The RNAPII signal is distributed unevenly along the bivalents (D1–D10). An SCAS is localized to one of the longest SCs (D9). **E.** Mid pachytene. Within an SCAS, there are separate RNAPII foci (E1,E2). Most bivalents have completed synapsis. Nonetheless, many bivalents have asynaptic "forks" (E3,E4). Intense RNAPII foci are noticeable throughout the nucleus. **F, G.** Early and mid diplotene. Desynaptic regions of chromosomes are visible. Some bivalents begin desynapsis from the terminal segments (yellow asterisks), and others from interstitial segments (blue asterisks). Pink asterisks indicate SCASs. Intense RNAPII foci are still present in the nucleus. **H.** Synapsis and desynapsis of some SC bivalents at different stages of prophase I (1, 1'–4, 4'). Scale bars = 5 μm (A–G).

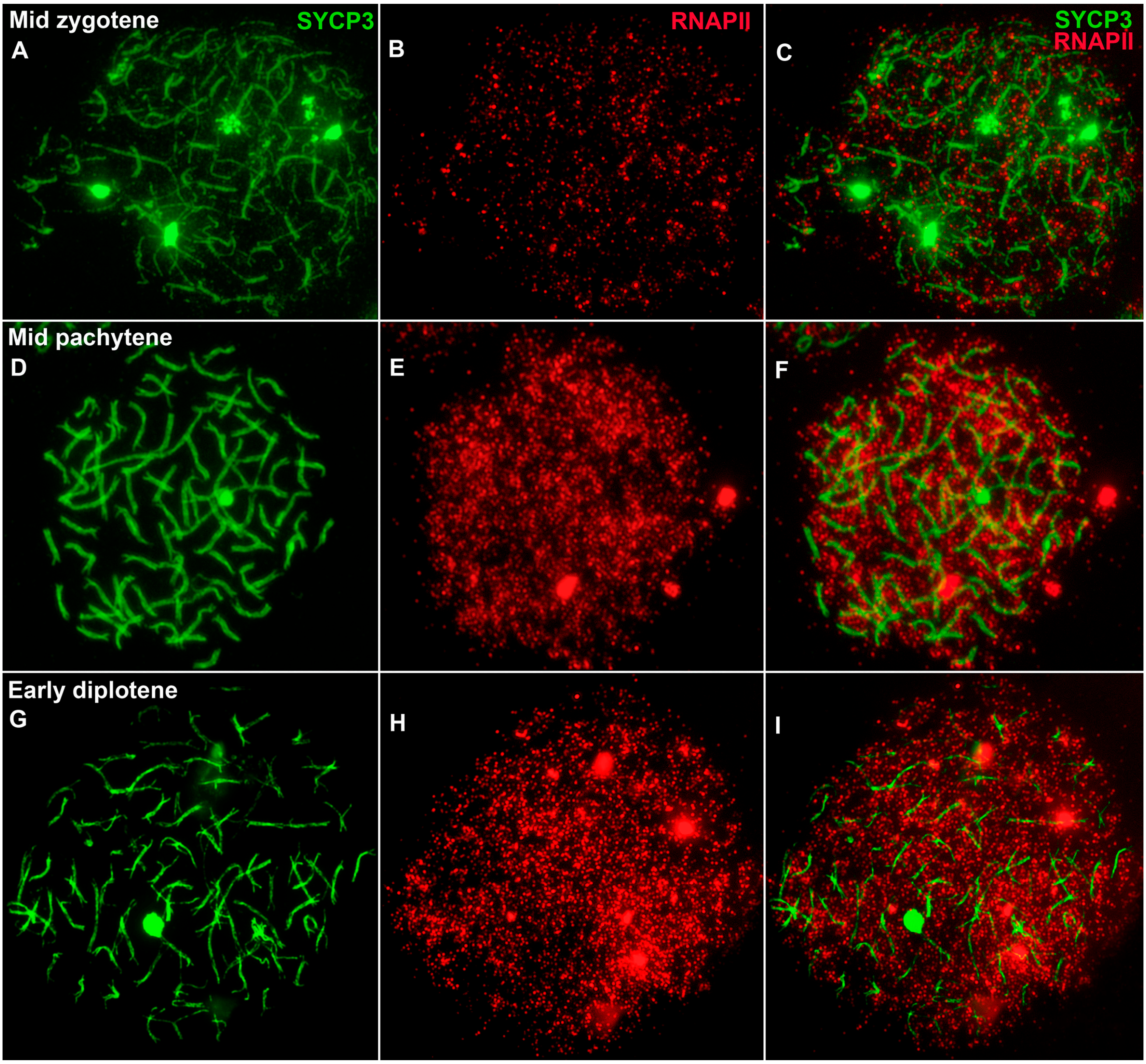

Figure S7. Immunolocalization of SYCP3 (green) and RNA polymerase (RNAPII, red) proteins in lamprey spermatocytes at the prophase I. **A-C.** Mid zygotene (see Fig. 1D); **D-F.** Mid pachytene; **G-I.** Early diplotene (see Fig. 2J).

Table S2. The average mean of fluorescence intensity of RNAPII signals per nucleus area (%) in lamprey spermatocytes at different substages of prophase I

	Mid zygotene	Late zygotene	Early pachytene	Mid pachytene	Diplotene
	A	B	C	D	E
Number of nuclei	20	28	31	32	22
Minimum	0,942	16,99	28,88	36,57	40,49
Maximum	9,909	48,98	70,13	85,9	72,54
Range	8,967	31,99	41,25	49,34	32,06
**Mean**	**5,474**	**30,04**	**45,94**	**64,09**	**53,64**
Std. Error of Mean	0,6338	1,825	1,819	2,58	2,386
Significant (P < 0.05)	A/B, A/C, A/D, A/E, B/C, B/D, B/E, C/D, C/E, D/E				

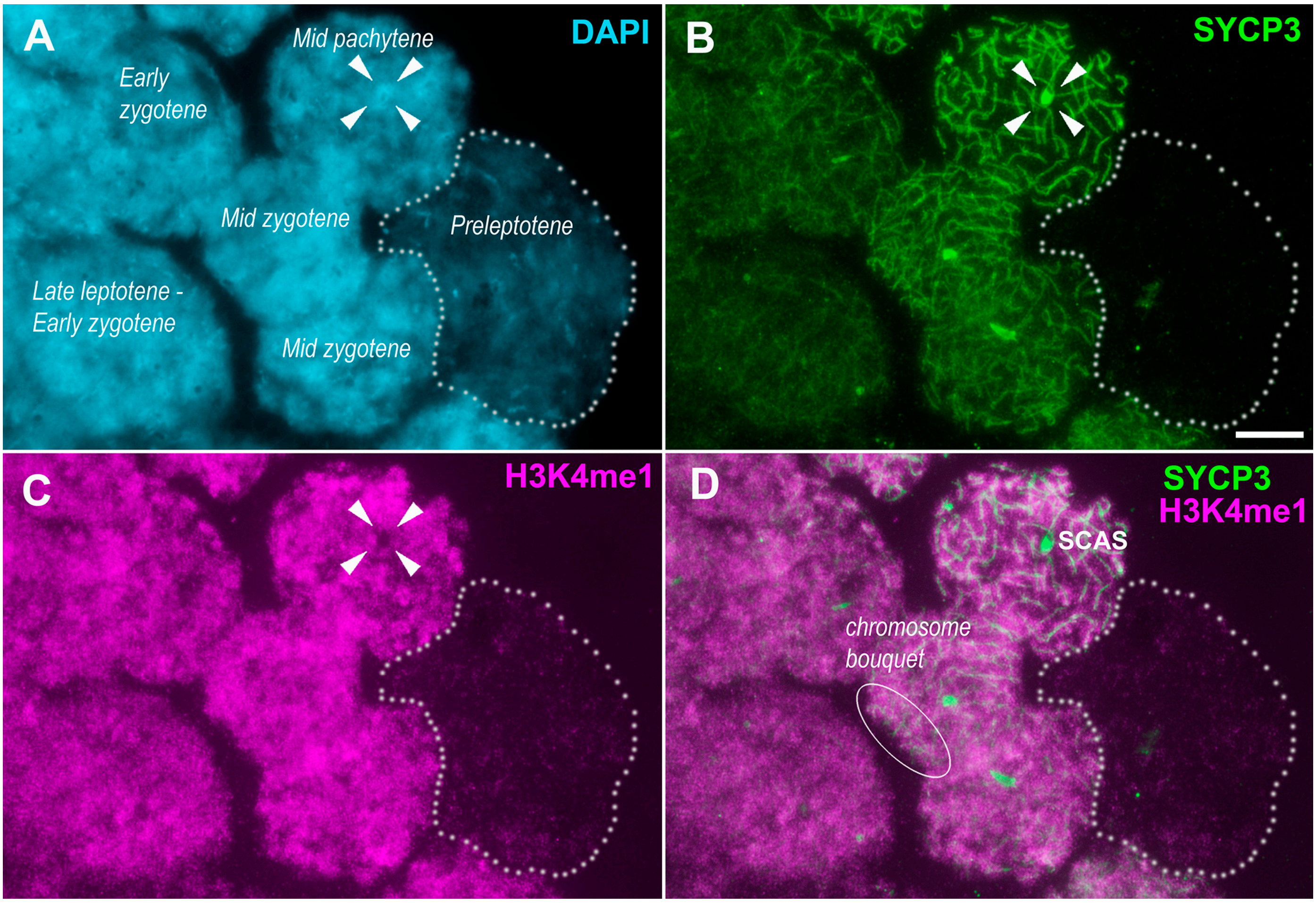

Figure S8. Immunolocalization of SYCP3 (green) and H3K4me1 (magenta) proteins in lamprey spermatocytes at different stages of prophase I. Dots indicate the preleptothene stage. Arrowheads indicate the SCAS. **A.** DAPI; **B.** SYCP3; **C.** H3K4me1; **D.** SYCP3 and H3K4me1. Scale bars = 5 μm

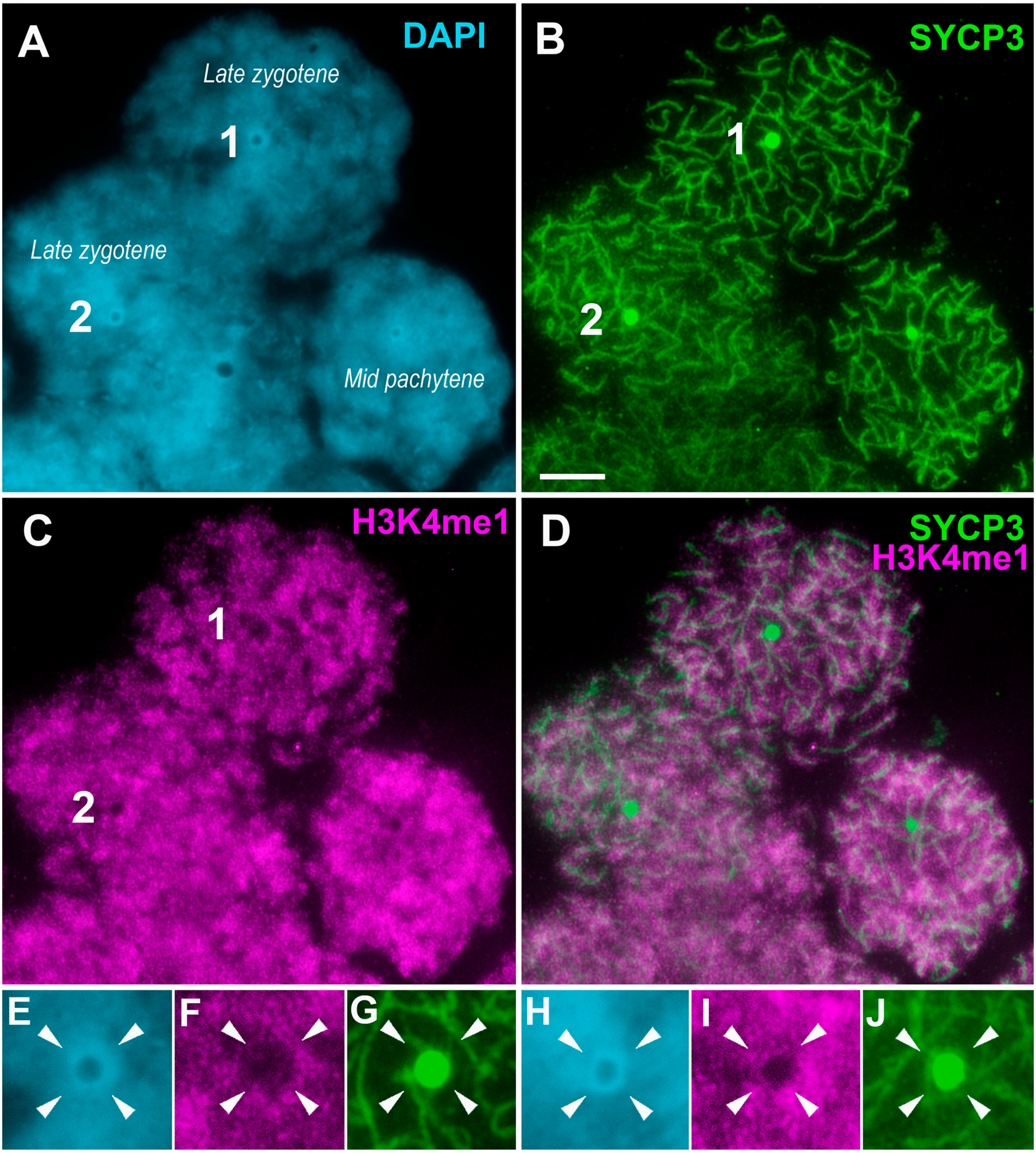

Figure S9. Immunolocalization of SYCP3 (green) and H3K4me1 (magenta) proteins in lamprey spermatocytes at different stages of prophase I. Arrowheads indicate the SCAS. **A.** DAPI; **B.** SYCP3; **C.** H3K4me1; **D.** SYCP3 and H3K4me1. **E-J.** Enlarged areas of nuclei (white numbers in A-C). Scale bars = 5 µm

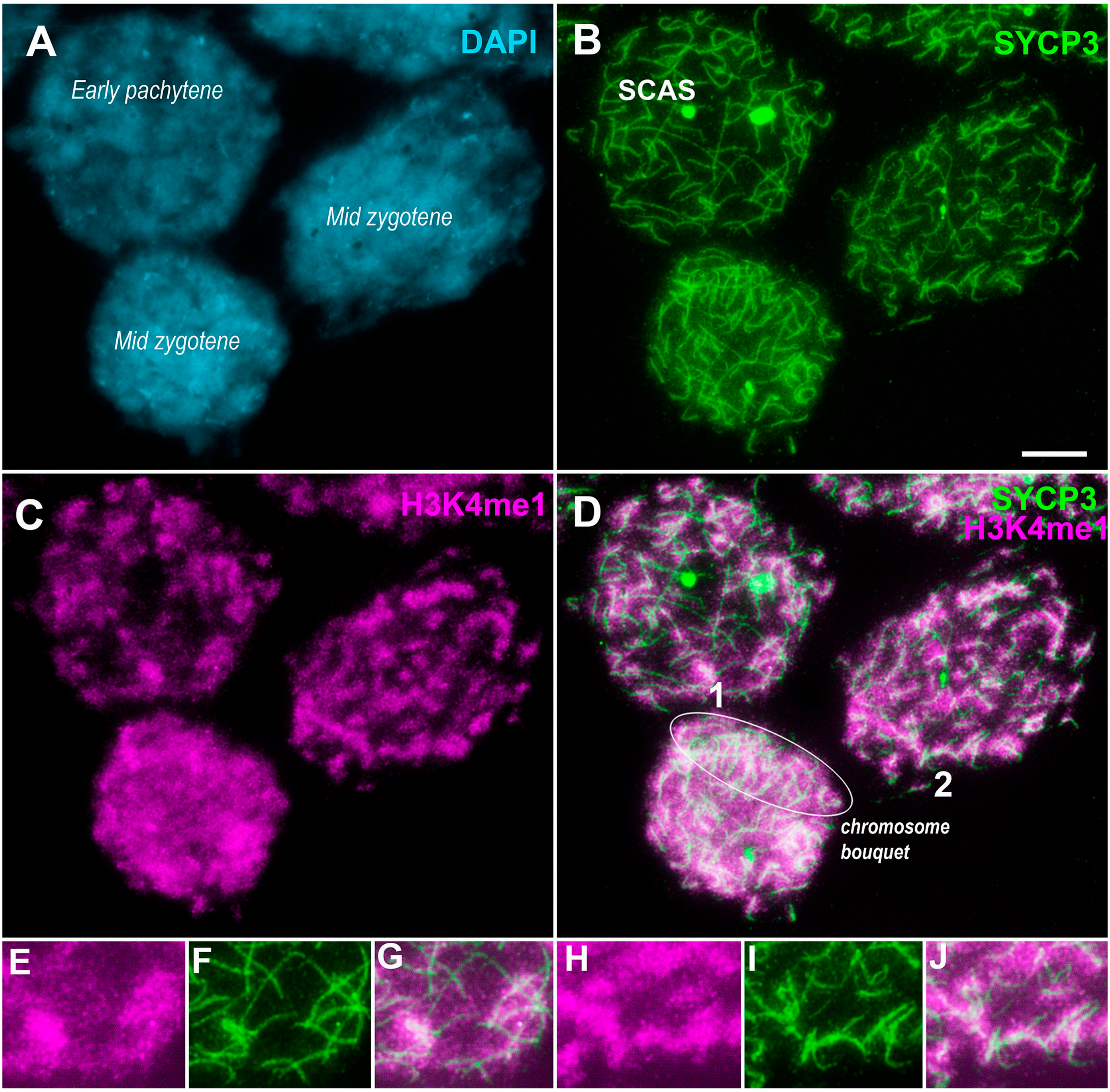

Figure S10. Immunolocalization of SYCP3 (green) and H3K4me1 (magenta) proteins in lamprey spermatocytes at different stages of prophase I. SCAS: an SC-associated structure in a long SC. **A.** DAPI; **B.** SYCP3; **C.** H3K4me1; **D.** SYCP3 and H3K4me1. **E-J.** Enlarged areas of nuclei (white numbers in D). Scale bars = 5 μm

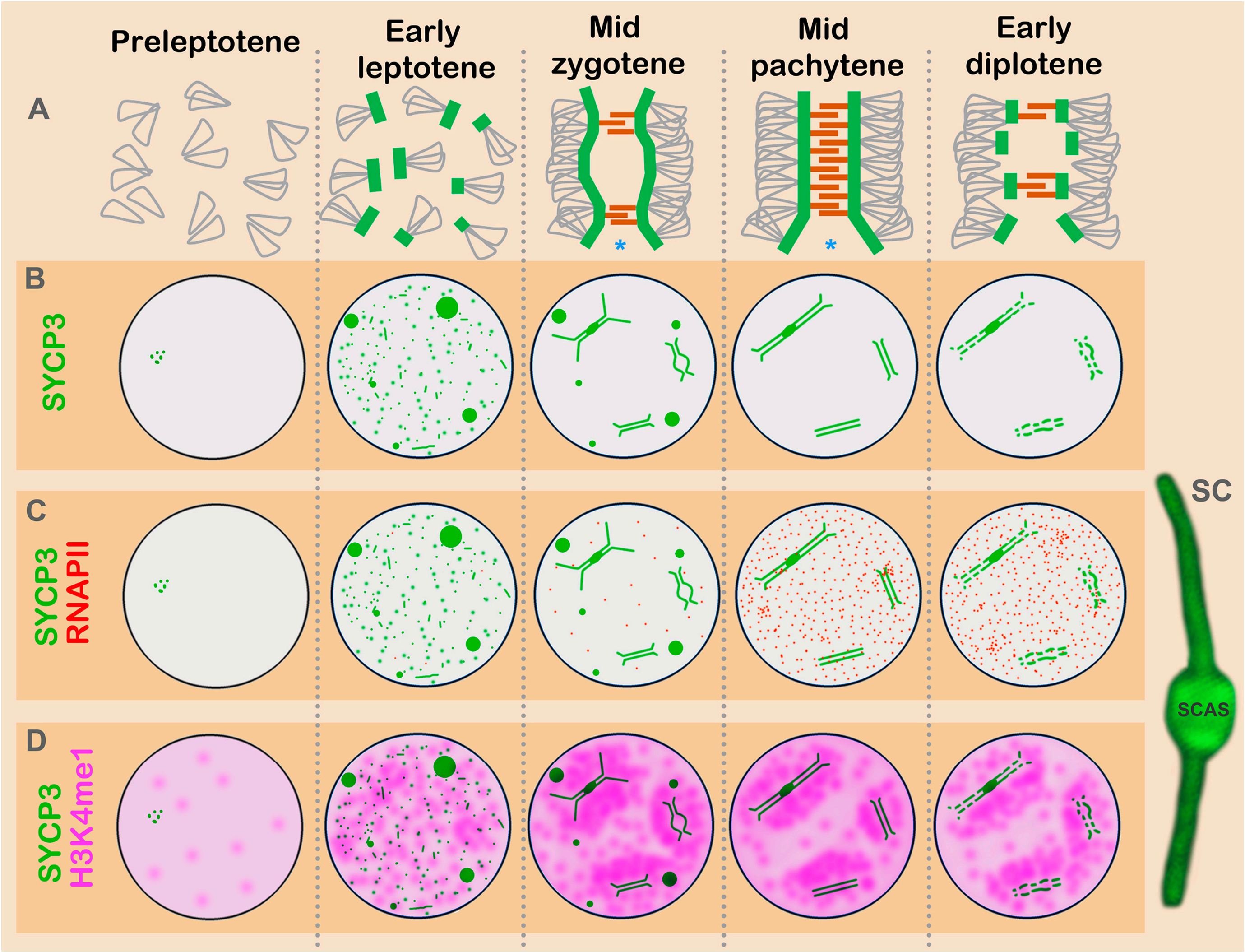

Figure S11. The meiotic first prophase in European river lamprey *Lampetra fluviatilis*. This scheme reflects the features of chromosome synapsis and distributions of some chromatin markers but does not show specifics of the interaction between SCs and the nuclear envelope. SCAS - SC-associated structure. **A.** Schematic representations of SCs at different prophase I substages. The green thick lines represent the lateral elements. The brown lines imitate transverse filaments of the central element of an SC. The gray lines depict chromatin loops. The blue asterisks denote telomeric asynaptic forks. **B.** Dynamics of axial or lateral (SYCP3) elements of SCs. **C.** Dynamics of SCs and of a transcriptional marker (RNAPII). **D.** Dynamics of SCs and of the H3K4me1 histone.
